# Implementation and validation of a commercial portal dosimetry software for intensity-modulated radiation therapy pre-treatment verification

**DOI:** 10.4103/0971-6203.71758

**Published:** 2010

**Authors:** C. Varatharaj, Eugenia Moretti, M. Ravikumar, Maria Rosa Malisan, Sanjay S. Supe, Renato Padovani

**Affiliations:** 1 Department of Medical Physics, University Hospital, Udine - 33100, India; 2 Department of Radiation Physics, Kidwai Memorial Institute of Oncology, Hosur Road, Bangalore - 560 029, India

**Keywords:** 2D-array detector, Electronic portal imaging device, intensity-modulated radiotherapy, portal dosimetry

## Abstract

Electronic portal imaging devices (EPIDs) are extensively used for obtaining dosimetric information of pre-treatment field verification and *in-vivo* dosimetry for intensity-modulated radiotherapy (IMRT). In the present study, we have implemented the newly developed portal dosimetry software using independent dose prediction algorithm EPIDose^™^ and evaluated this new tool for the pre-treatment IMRT plan quality assurance of Whole Pelvis with Simultaneous Integrated Boost (WP-SIB-IMRT) of prostate cases by comparing with routine two-dimensional (2D) array detector system (MapCHECK^™^). We have investigated 104 split fields using γ -distributions in terms of predefined γ frequency parameters. The mean γ values are found to be 0.42 (SD: 0.06) and 0.44 (SD: 0.06) for the EPIDose and MapCHECK^™^, respectively. The average γ∆ for EPIDose and MapCHECK^™^ are found as 0.51 (SD: 0.06) and 0.53 (SD: 0.07), respectively. Furthermore, the percentage of points with γ < 1, γ < 1.5, and γ > 2 are 97.4%, 99.3%, and 0.56%, respectively for EPIDose and 96.4%, 99.0% and 0.62% for MapCHECK^™^. Based on our results obtained with EPIDose and strong agreement with MapCHECK^™^, we may conclude that the EPIDose portal dosimetry system has been successfully implemented and validated with our routine 2D array detector

## Introduction

The clinical implementation of intensity-modulated radiotherapy (IMRT) requires the ability to verify complex radiation beams. However, a practical drawback on the implementation of IMRT into the clinical routine remains the time-consuming patient-specific quality assurance (QA) that precedes the actual treatment. The most widely used form of pre-treatment QA for IMRT generally consists of absolute dose measurements (with ionization chamber, thermo luminescent dosimeter, etc.) combined with planar dose distribution measurements in a phantom. The phantom has to be equipped with dose measuring devices such as radiochromic films, two-dimensional (2D) ion chamber or diode-based detectors. Even though the film dosimetry is widely accepted due to its spatial resolution, doing QA with film is a time-consuming process due to phantom setup, film development, and film analysis and also there are issues with uncertainties due to processor artifacts. Ion chamber and diode array allow for fast analysis,[1–4] but they both have a reduced data density compared to film. In addition, ion chambers exhibit marked volume averaging that requires the treatment planning system (TPS) calculated dose to be blurred prior to IMRT QA.[5,6]

Electronic portal imaging devices (EPIDs) have replaced the conventional radiographic films for the acquisition of portal images in radiotherapy. EPIDs were originally implemented for patient position verification, but their use has been later extended to obtain dosimetric information for pre-treatment field verification and *in vivo* dosimetry.[7–9] Applications in this field have been performed to predict portal dose images for portal imaging devices of various types. A practical advantage of EPID-based dosimetric verification is its availability and simplicity of use. There is neither phantom nor additional connection of devices necessary. In literature there are many reports on different type of portal dosimetry systems such as fluoroscopy-based systems,[10,11] liquid filled ionization chamber matrices-based[12,13] and amorphous silicon (aSi)-based systems.[14,15] Over the last few years, aSi detectors have become increasingly popular for online portal imaging, requiring less excess dose to be delivered to the patient portal image and yet yielding a superior quality image than the liquid filled ionization chamber EPID.

Munro *et al*.[16] and Bouius investigated in detail the characteristics of a small (96 × 96 mm^2^) aSi flat panel detector.[16] They measured the linearity, spatial resolution, glare, noise, and signal-to-noise characteristics of an indirect aSi EPID construction, containing a metal plate/phosphor screen generating optical photons that are detected by the photodiodes. Greer and Popescu[17] investigated the dosimetric properties of an aSi EPID using a continuous frame-averaging acquisition mode and a 6 MV radiation beam. They concluded that the aSi EPID showed promise as an efficient verification tool for IMRT delivery, the main limitations being related to the dead time in the frame acquisition and sensitivity calibration. Van Esch *et al*.[15] explored the possibility of using a commercially available aSi portal imager for absolute dosimetric verification of the delivery of dynamic IMRT fields.

In the present study, we have implemented the newly developed portal dosimetry software using independent dose prediction algorithm (EPIDose^™^, Sun Nuclear Corporation, Melbourne, FL) and evaluated this new tool for the pre-treatment QA of whole pelvis with simultaneous integrated boost for step and shoot IMRT (WP-SIB-IMRT) of prostate cases. The software calculates the dose distribution in a phantom from the fluence image acquired by EPID. To validate it as a tool for routine QA, we compared the planar doses of all plans with our routine 2D-diode array detector MapCHECK^™^ (Sun Nuclear Corporation).

The use of new dosimetric tools and procedures for clinical QA practice is becoming increasingly important, especially when taking into account the necessity of reliable but also time-sparing QA protocols. Although each institution has developed its own equipment and modality, most of them use the γ index for QA analysis first presented by Low *et al*.[18] The efficacy of the IMRT technique and the consequently increasing number of treated patients, along with increased planning and delivery experience, assert the necessity of defining reliable pass/fail criteria to facilitate the dose distribution comparison process. The importance of this issue finds its confirmation in several published works, where the interpretation of the γ map is addressed as a crucial point.[19] The fluence analysis has now being established an essential tool to evaluate the consistency of two dose maps (measured and calculated). Its efficiency derives from the fact that it conjugates both dose difference (DD) and distance to agreement (DTA) pass/fail criteria for dose distribution comparisons. Hence, in this study, statistics of γ-values were used to quantify the agreement between measured dose distribution and calculated dose distribution in the validation of WP-SIB-IMRT plans. The main objective of the present work is to implement the newly developed portal dosimetry software EPIDose^™^ and evaluate this new tool for the pre-treatment IMRT plan QA of whole pelvis with simultaneous integrated boost (WP-SIB-IMRT) of prostate cases by comparing with routine 2D array detector system (MapCHECK^™^).

## Materials and Methods

All measurements were performed using a 6 MV X-ray beam from a Clinac 600C linear accelerator (Varian Associates, Palo Alto, CA, USA) equipped with Millennium120 multileaf collimator (MLC). MLC contains 120 leaves; it is designed with 5-mm width leaf (central 20 cm of field) and 10-mm width leaf (outer 20 cm of field) projected at isocenter. The maximum over travel across the beam axis is 16 cm, and the maximum distance from a leaf to the carriage is 15 cm. Due to this limitation, for large target volumes (as in the case of Whole Pelvis) it is necessary to split the fields into 2 parts. In this work, we have compared the measured doses between the two detectors (EPIDose^™^ and MapCHECK^™^) and TPS calculated ones for every split field.

### The aSi 500 EPID system

All EPID images were acquired with an aSi-500 imaging device mounted on a linear accelerator. The EPID system includes (1) image detection unit (IDU), featuring the aSi detector and accessory electronics; (2) image acquisition system 2 (IAS3) containing acquisition electronics for the IDU and interfacing hardware; and (3) a Portal Vision workstation. Within the detector, a scintillator converts the incoming X-rays into visible photons. The phosphor scintillator converts incident radiation into optical photons, enhancing the sensitivity of the detector more than tenfold.[6,12] The light is sensed by a photodiode array attached to the amorphous silicon panel. The photodiodes integrate the incoming light into charge captures, and the detector electronics transfer the charges from pixels to read-out electronics. The sensitive area of the panel is 512 × 384 pixels, with a pixel size of 0.784 mm and a total sensitive area of 40 × 30 cm^2^. The intrinsic water equivalent thickness is 8 mm as per manufacturer specification. In the present work, we acquired all images in the “integrated mode”.

EPID is calibrated by the acquisition of dark field (DF) and flood field (FF) images as per the vendor instructions. The DF image (formed by 100 frames) is acquired with no radiation and records the pixel offsets. The FF image (formed by 200 frames) is recorded with an open field uniform irradiation covering EPID area to determine the differences in individual pixel sensitivities. The defective pixels showing very cold or hot signals are automatically removed from the EPID by applying a pre-determined defect map obtained by the portal vision software through a drift image.[20] The basic characteristics of aSi EPID dosimetric performance were extensively analyzed in the initial phase of our study and the results are comparable with the published papers.[15] All the measurements have been performed at a source to detector distance of 105 cm and gantry at 0°. Before acquiring EPID image of every field, a dark field image was acquired.

### The MapCHECK™ - 2D diode array system

The two-dimensional dose measuring device used in this study is the MapCHECK™ Model 1175 (Sun Nuclear). The system consists of 445 n-type diodes that are in a 22 × 22 cm^2^ 2-D array with variable spacing between diodes. The matrix represents two detector densities; those in the central detection area (10 × 10 cm^2^) contains 221 diodes spaced 10 mm and each line of detectors is shifted 5 mm with respect to the next, in such manner the diagonal spacing between detectors is 7.07 mm; in the outer part there are 221 diodes spaced 20 mm and each line is shifted 10 mm. In this way, the resulting diagonal spacing is 14.14 mm. Each detector has an active area of 0.8 × 0.8 mm^2^. The proprietary design of these n-type diodes makes them resistant to damage by radiation. Two acrylic plates that have conductive surfaces envelop the diodes, which are mounted on a multilayered circuit board. This provides shielding from radio frequency fields generated in a linear accelerator. The inherent buildup of the instrument is 2 g/cm^2^, while the linear depth from the top of the detection plane is 1.35 cm and the inherent backscatter thickness is 2.27 g/cm^2^.

Each detector is connected to the input of a low leakage, high gain metal-oxide-semiconductor field-effect transistor (MOSFET) operational amplifier, which integrates the signal during irradiation. Signal processing is done by a personal computer connected through an amplifier interface circuit. A diode-relative-sensitivity calibration procedure, performed with a built-in software application, determines the sensitivity of each diode with respect to the central diode. For measurements of the present work, plane of the diodes is at 100 cm from the accelerator radiation source and the diodes are at a water equivalent depth of 5 g/cm^2^. This is provided by 3 cm of solid water on top of the MapCHECK^™^ with its intrinsic thickness of 2 g/cm^2^, 1.35 cm physical thickness, of buildup. Initially, we have analyzed the basic dosimetric characteristics of MapCHECK^™^, but they are not discussed here, since they are widely reported in literature.[1,19]

### Conversion of EPID image using EPIDose^™^ software

EPIDose^™^ is a commercial software application (Sun Nuclear, Melbourne, FL, USA), which allows to convert the image acquired by an EPID in a dose map and to compare the dose map with a reference dose distribution. For the purpose of implementation of EPIDose^™^, a set of integrated images of open fields of different intensities and sizes are acquired and consequently imported into EPIDose^™^ together with the output factor table measured by MapCHECK^™^ to establish basic algorithm configuration data. The processes carried out by the EPIDose^™^ algorithm are specified in four steps.[21] In the first step, a simple geometric back-projection technique is applied to the raw EPID image to scale its pixels to the desired dose plane distance. Second, corrections for output factors and the source distribution of scattered photons are applied to each MLC sub-fields. In the third step, the results are convolved with a dose redistribution kernel to simulate wider electron spread in water. In the final step, the relative dose maps are then converted to dose using calibration array data in the EPIDose^™^ model based on MapCHECK^™^ absolute dose planes. As a first part of clinical commissioning of EPIDose^™^, we have validated its performance by comparing the measurements of simple fields as square, rectangular fields and off-axis fields with MapCHECK^™^. we adopted the following γ-criteria: 2 mm and 2%.

### IMRT plan details

Recently, in our institution we have started step and shoot IMRT (S & S IMRT) treatments for WP-SIB to prostate (25 fractions) using the optimization module (Direct Step and Shoot) of Masterplan TPS (Nucletron, Veenendaal, The Netherlands). The algorithm of dose was enhanced pencil beam. The prescribed doses were 67.5 Gy, 56.5 Gy, and 50 Gy delivered to prostatic gland, seminal vesicles, and pelvic lymph nodes, respectively.

Our routine pre-treatment patient specific QA includes 2D planar doses verification and absolute point dose. For the first type of QA, we employ MapCHECK^™^ matrix measuring the dose distribution of each split field. The comparison between calculated and measured dose is made by means of the gamma function analysis,[18] adopting a DD criterion of 3% and a DTA criterion of 3 mm. For the calculation of γ-index, we use the analysis tool of Mapcheck^™^ software. The γ-index is calculated in local dose. We exclude from the analysis the points having a dose value lower than the 10% of the maximum dose. A plan is accepted if the percentage of points with gamma below 1 is higher than 90%. The point dose measurements were carried out using a calibrated 0.6 cm^3^ Farmer-type ionization chamber (NE2571, Nuclear Enterprises, Fairfield, NJ) to assess the dose in a clinically meaningful high-dose gradient region, delivering all beams at original gantry angle. As a phantom, we employed a polymethyl methacrylate (PMMA) slab phantom. The acceptance criterion for point dose measurement is a maximum deviation of 3% between calculated and measured value.

For the clinical commissioning of EPIDose^™^, we have considered a total number of 104 split fields of 10 different WP-IMRT plans using both EPIDose^™^ and MapCHECK^™^. The treatment configurations were as follows: one including 5 beams (n = 9 patients with 10 splitted fields per patient) and the second one consisting in 7 beams (n = 1 patient with 14 splitted fields). We obtained an improvement of the spatial resolution of the diode matrix device by irradiating it in different position of the same field and merging the resultant dose images.

### Dosimetric evaluation using γ index analysis tool

The planar map of γ-values gives a qualitative representation of the agreement between two dose distributions, but it must be evaluated further in terms of its acceptability. For this map to serve as an efficient pass/fail criteria in a QA comparison procedure it would be preferable that it satisfies some uniquely established quantitative criteria. The γ-area histograms, defining the percentage of γ values below a certain threshold, can lead to the definition of these criteria.[22] In the current investigation, γ value distribution for 104 split fields were analyzed using γ distributions. In particular, for each split field the following γ distribution parameters were determined:[23]

- mean γ value.
- γ_∆_ value, defined as the previous mean γ value + 1.5 SD.
- Percentage of points with γ < 1, γ < 1.5, and γ > 2.

The γ values were calculated by the analyzing software of MapCHECK^™^.

## Results

**Figure 1:**
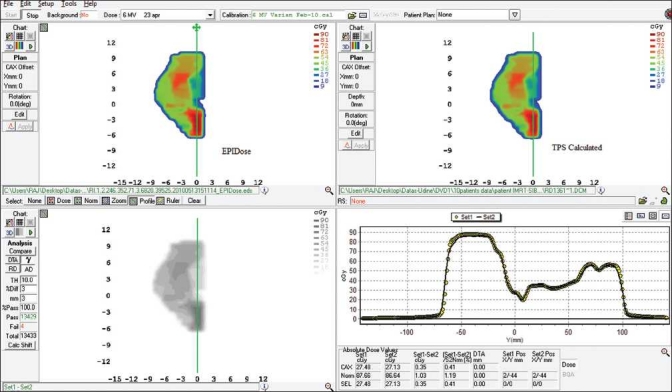
Comparison of results between the EPIDose and TPS calculated one for a split field of a typical prostate case using 3 mm and 3% criteria. Comparison of profiles along the Y-axis is shown in the lower right window. The mean γ value and percentage of points with γ < 1 are 0.42 (SD: 0.06) and 100%, respectively

**Figure 2:**
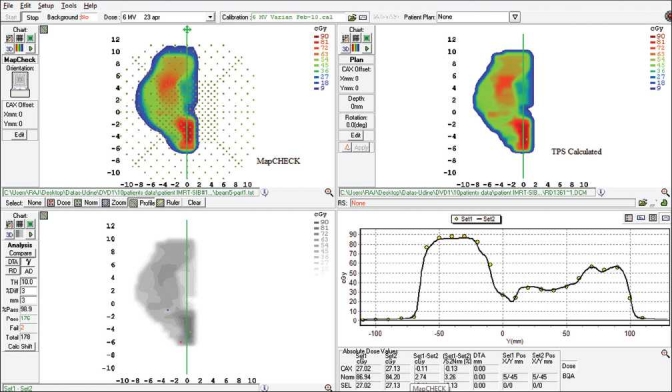
Comparison of results between the MapCHECK measured and TPS calculated one for a split field of a typical prostate case using 3 mm and 3% criteria. Comparison of profiles along the Y- axis is shown in the lower right window. The mean γ value and percentage of points with γ < 1 are 0.47 (SD: 0.08) and 98.9%, respectively

**Figure 3:**
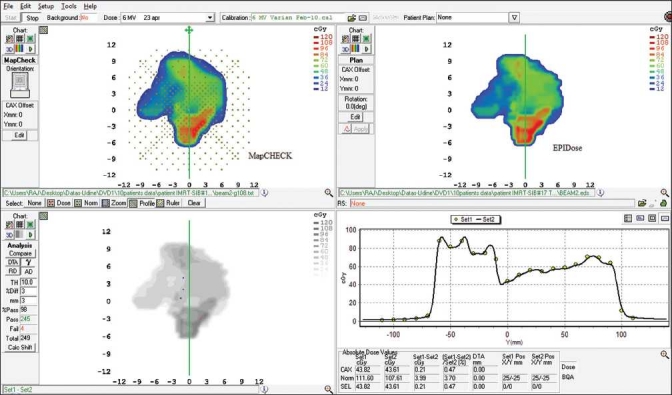
Comparison of results between the MapCHECK measured and EPIDose for a merged field of a typical prostate case using 3 mm and 3% criteria. Comparison of profiles along the Y- axis is shown in lower right window. The mean γ value and percentage of points with γ < 1 are 0.52 (SD: 1.2) and 98.0%, respectively

Figure 1shows a sample of comparison of measured dose maps using EPIDose^™^ with TPS calculated doses. The comparison of profiles along the Y-axis is shown in lower right window. A similar example of comparison between the MapCHECK ^™^ measured and TPS calculated doses is shown in Figure 2. The comparison between MapCHECK^™^ and EPIDose^™^ for a merged field (i.e., merging 2 split fields) is shown in Figure 3. The measured fields using EPIDose^™^ in terms of percentage of points with γ ≤ 1 resulted in well agreement with MapCHECK^™^ measured fields.

The values of the γ frequency parameters (namely, mean γ, γ_∆_, and the percentage of points with γ < 1, γ < 1.5, and γ > 2) obtained from the cumulative histogram statistical analysis for the WP-IMRT 104 fields corresponding to ten different patients are summarized in Table 1 both for EPIDose^™^ and MapCHECK^™^ measurements. The mean γ values are determined to be 0.42 (SD: 0.06) and 0.44 (SD: 0.06) for EPIDose^™^ and MapCHECK^™^, respectively. The average γ for EPIDose^™^ and MapCHECK^™^ are determined to be 0.51 (SD: 0.06) and 0.53 (SD: 0.07), respectively. Furthermore, the percentage of points with γ < 1, γ < 1.5, and γ > 2 are 97.4%, 99.3%, and 0.56%, respectively for EPIDose^™^ and 96.4%, 99.0%, and 0.62% for MapCHECK^™^.

**Table 1:** Comparison of statistical parameters used to define operative criteria for the pass/fail agreement procedures between MapCHECK^™^ and EPIDose

*Patient no.*	*No. of. fields analyzed*	*Average γ value*		*Average γ_∆_ value*		*Average percentage of points with γ < 1*		*Average percentage of points with γ < 1.5*		*Average percentage of points with γ > 2*	
		*MapCHECK^™^*	*EPIDose^™^*	*MapCHECK^™^*	*EPIDose^™^*	*MapCHECK^™^*	*EPIDose^™^*	*MapCHECK^™^*	*EPIDose^™^*	*MapCHECK^™^*	*EPIDose^™^*
1.	10	0.44	0.40	0.54	0.49	97.7	98.1	99.6	99.3	0.4	0.7
2.	10	0.50	0.42	0.61	0.55	95.5	96.6	99.2	99.4	0.5	0.5
3.	10	0.60	0.39	0.69	0.44	93.5	98.4	98.9	99.5	1.0	0.4
4.	14	0.37	0.38	0.46	0.46	98.4	97.6	99.5	98.9	0.4	0.9
5.	10	0.41	0.40	0.52	0.49	97.7	97.4	99.1	99.5	1.2	0.4
6.	10	0.44	0.52	0.54	0.63	95.9	94.8	98.1	99.0	0.8	0.6
7.	10	0.42	0.39	0.50	0.51	97.1	97.5	99.1	99.2	0.5	1.0
8.	10	0.41	0.41	0.47	0.46	96.9	98.9	99.3	99.7	0.2	0.2
9.	10	0.40	0.45	0.45	0.54	96.5	96.2	99.3	98.7	0.2	0.7
10.	10	0.45	0.41	0.56	0.52	94.9	98.9	98.3	99.7	1.0	0.2
Mean		0.44	0.42	0.53	0.51	96.4	97.4	99.0	99.3	0.62	0.56
SD		0.06	0.06	0.07	0.06	2.12	1.70	0.95	0.75	0.84	0.72

**Figure 4:**
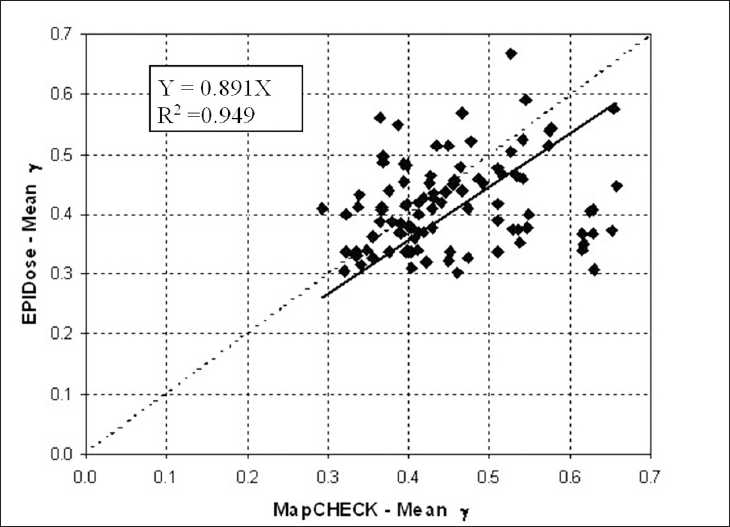
Linear regression analysis between MapCHECK and EPIDose in terms of mean γ values using 3 mm and 3% criteria for the 104 analyzed fields. The dotted diagonal line represents the ideal match between the data. The inner legend shows the correlation equation and coefficient

**Figure 5:**
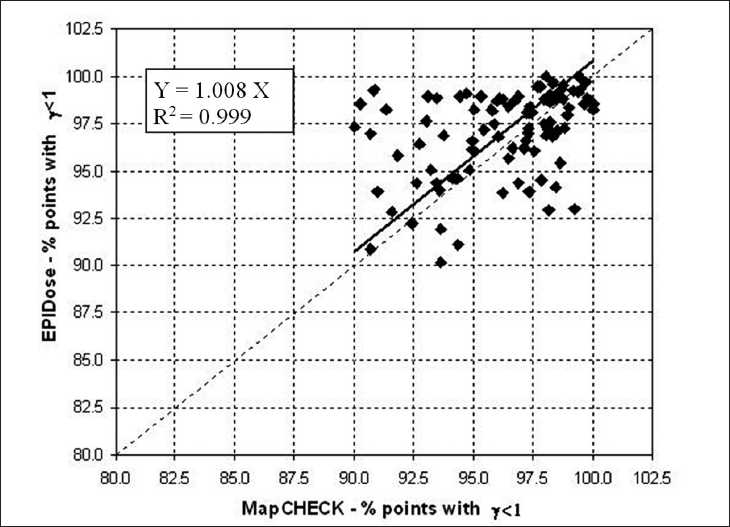
Linear regression analysis between MapCHECK and EPIDose in terms of percentage of points with γ < 1 using 3 mm and 3% criteria for the 104 analyzed fields. The dotted diagonal line represents the ideal match between the data. The inner legend shows the correlation equation and coefficient

**Figure 6:**
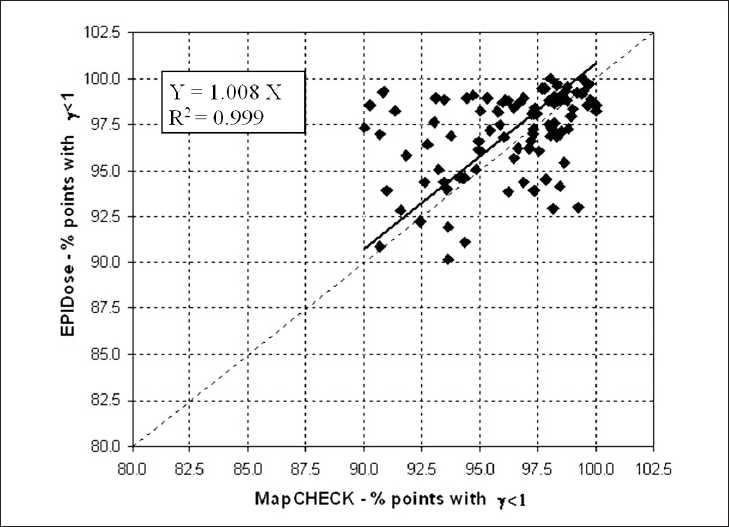
Linear regression analysis between MapCHECK and EPIDose in terms of percentage of points with γ < 1.5 using 3 mm and 3% criteria for the 104 analyzed fields. The dotted diagonal line represents the ideal match between the data. The inner legend shows the correlation equation and coefficient

We have quantified the agreement between the two dosimetric verification methods by determining the correlation of the two set of results. For statistical analysis, we applied the linear regression test by Pearson (SYSTAT 10, Systat Software, Inc., Chicago, IL, USA). Figures 4, 5, and 6 show the statistical analysis between the overall values obtained from the measurements of two detector systems in terms of mean γ, percentage of points with γ < 1 and γ < 1.5, respectively. The correlation coefficient were 0.949, 0.999, and 1.000, respectively for the repopulation of mean γ, percentage of points with γ < 1 and γ < 1.5.

## Discussion and Conclusions

The aim of this study is to implement and validate the EPIDose^™^ portal dosimetry system as a routine QA tool for patient specific pre-treatment verification. We have analyzed the statistical analysis of γ values to identify the agreement between measured dose distribution and calculated dose distribution in the validation of the WP-SIB-IMRT plans for EPIDose^™^ and with our routine QA tool MapCHECK^™^. Due to high level of efficiency, the γ index is the tool of choice for the analysis of results from IMRT verification, and an increasing number of published works state that γ histograms may provide a valuable evaluating method for dose distribution comparison. Spezi *et al*.[22] suggested that the γ-area histograms are extremely useful in visually locating regions wherein significant discrepancies occur. Other papers dealing with the similar statistical analysis of pre-treatment dosimetry can be found in literature.[24–26]

We evaluated the dosimetric performance of EPIDose^™^ by comparing it with MapCHECK^™^; MapCHECK^™^being our standard tool for patient-specific QA in IMRT. The comparison was made in terms of the agreement parameters mean g, mean g_∆_, percentage of points with γ < 1, γ < 1.5, and γ > 2.

The mean γ, mean γ_∆_ values, the percentage of points with γ < 1, γ < 1.5, and γ > 2 values for EPIDose^™^ were in good agreement with MapCHECK^™^ estimated values. The correlation graphs of the Figures 4, 5, and 6 show a slight trend towards better results with EPIDose^™^ than MapCHECK^™^ dosimetry, i.e., lower mean γ values, higher percentage of points with γ < 1 and similar trend for percentage of points with γ < 1.5.

In general, the obtained results with both EPIDose^™^ and MapCHECK^™^ are comparable. The slightly better values of the γ parameters obtained using EPIDose^™^ could be due to its finer spatial resolution.

Therefore, based on our results obtained with EPIDose^™^ and strong agreement with MapCHECK^™^, we may conclude that the EPIDose^™^ portal dosimetry system has been successfully implemented and validated with our routine 2D array detector for Step-and-Shoot IMRT of prostate cases. Furthermore, portal dosimetry using EPIDose^™^ is an efficient and fast method for a routine verification of intensity modulated fields.
